# Measurement properties of the EQ-5D-5L compared to the EQ-5D-3L across eight patient groups: a multi-country study

**DOI:** 10.1007/s11136-012-0322-4

**Published:** 2012-11-25

**Authors:** M. F. Janssen, A. Simon Pickard, Dominik Golicki, Claire Gudex, Maciej Niewada, Luciana Scalone, Paul Swinburn, Jan Busschbach

**Affiliations:** 1 Department of Medical Psychology and Psychotherapy, Erasmus MC, Erasmus University, PO Box 2040, 3000 CA Rotterdam, The Netherlands; 2 Center for Pharmacoeconomics Research, College of Pharmacy, University of Illinois at Chicago, 833 South Wood St, MC886, Chicago, IL USA; 3 HealthQuest, Wyspiańskiego 4/5, 01-577 Warsaw, Poland; 4 Department of Endocrinology, Osteoporosis Clinic, Odense University Hospital, Kløvervænget 6, 1st floor, 5000 Odense C, Denmark; 5 Department of Experimental and Clinical Pharmacology, Medical University of Warsaw, Krakowskie Przedmieście 26/28, 00-927 Warsaw, Poland; 6 Research Centre on Public Health, University of Milano Bicocca, Villa Serena, Via Pergolesi 33, 20052 Monza, Italy; 7 CHARTA Foundation, Milan, Italy; 8 Oxford Outcomes (ICON plc), Searcourt Tower, West Way, Oxford, OX2 0JJ UK

**Keywords:** EQ-5D, Health-related quality of life, Psychometrics, Patient-reported outcomes, Utility assessment

## Abstract

**Purpose:**

The aim of this study was to assess the measurement properties of the 5-level classification system of the EQ-5D (5L), in comparison with the 3-level EQ-5D (3L).

**Methods:**

Participants (*n* = 3,919) from six countries, including eight patient groups with chronic conditions (cardiovascular disease, respiratory disease, depression, diabetes, liver disease, personality disorders, arthritis, and stroke) and a student cohort, completed the 3L and 5L and, for most participants, also dimension-specific rating scales. The 3L and 5L were compared in terms of feasibility (missing values), redistribution properties, ceiling, discriminatory power, convergent validity, and known-groups validity.

**Results:**

Missing values were on average 0.8 % for 5L and 1.3 % for 3L. In total, 2.9 % of responses were inconsistent between 5L and 3L. Redistribution from 3L to 5L using EQ dimension-specific rating scales as reference was validated for all 35 3L–5L-level combinations. For 5L, 683 unique health states were observed versus 124 for 3L. The ceiling was reduced from 20.2 % (3L) to 16.0 % (5L). Absolute discriminatory power (Shannon index) improved considerably with 5L (mean 1.87 for 5L versus 1.24 for 3L), and relative discriminatory power (Shannon Evenness index) improved slightly (mean 0.81 for 5L versus 0.78 for 3L). Convergent validity with WHO-5 was demonstrated and improved slightly with 5L. Known-groups validity was confirmed for both 5L and 3L.

**Conclusions:**

The EQ-5D-5L appears to be a valid extension of the 3-level system which improves upon the measurement properties, reducing the ceiling while improving discriminatory power and establishing convergent and known-groups validity.

## Introduction

As a generic preference-based measure of health, the EQ-5D has many applications that aid decision making in health care [1]. The standard format of the EQ-5D descriptive classification system developed by the EuroQoL Group consists of five dimensions of health, each with three levels of problems (EQ-5D-3L, hereafter “3L”). Over the past twenty years, value sets for the 3L health classification system have been developed for many countries around the world [2].

There is an extensive body of literature to support the validity and reliability of the 3L descriptive system, the EQ-VAS, and the 3L index values in many conditions and populations [3–8]. However, its restricted ability to discriminate small to moderate differences in health status has been questioned widely [9–12]. Moreover, several studies reported a ceiling effect of the 3L in both general population and patient settings [13–18].

The EuroQol Group has recently introduced a 5-level EQ-5D (EQ-5D-5L, hereafter “5L”), which expands the range of responses in each dimension from three to five levels [19]. Preliminary studies indicated that prototype 5L versions improved upon the properties of the 3L in terms of reduced ceiling effects, increased reliability, and improved ability to discriminate between different levels of health [20–22].

A Korean study has shown good measurement properties for the 5L in cancer patients [23]. To our knowledge, there has been no validation of other language versions of EQ-5D-5L, nor has there been assessment of measurement properties in other patient groups or a combination of patients groups. The goal of this study was to assess the measurement properties of the 5L, in comparison with the 3L, across a wide range of patient groups. The specific aims were to evaluate and compare the properties of 3L and 5L in terms of feasibility (missing values), consistent redistribution of responses from 3L to 5L, ceiling, discriminatory power (Shannon indices), convergent validity, and known-groups validity.

## Methods

### Data

This study aimed at assessing measurement properties for 3L and 5L in eight broad patient groups. A student cohort was added in order to investigate how both instruments perform in a healthy population sample. Respondents completed both the 3L and 5L in six countries: Denmark, England, Italy, the Netherlands, Poland, and Scotland. Data collection in Denmark was conducted through the endocrinology, rheumatology, and orthopedic departments of a regional university hospital. Data collection in England was organized through a specialist patient recruitment agency and aimed at patients with prespecified conditions. In Italy the cohort of liver disease patients completed the questionnaires locally at two hospitals (Bergamo and Naples). Data collection in the Netherlands was conducted at a specialist center for personality disorders and at a local hospital for the kidney dialysis patients. In Poland, the student cohort was recruited at the Medical University of Warsaw in Poland, and the stroke cohort was recruited through the Neurological Clinic in Warsaw. Data collection in Scotland took place through a specialist patient recruitment agency, with patients completing the questionnaires at primary care centers. Paper and pencil versions of the questionnaires were used in all countries except in England where data collection took place online. Data collection took place between August 2009 and September 2010. The 5L was administered first, followed by the EQ-5D visual analogue scale (EQ-VAS) and a number of demographic questions, then the 3L, and finally a set of five dimension-specific rating scales. All respondents scored 5L first, as a previous study showed a tendency to avoid the in-between levels 2 and 4 of 5L when responding to the 3L first [20]. Data collection was undertaken with informed consent and according to the ethical guidelines for health research in each country.

### Measures

The 3L version of the EQ-5D is the initial version that has been used in many clinical trials and methodological studies published in the peer-reviewed literature [1]. It is a brief self-reported generic measure of current health that consists of five dimensions (Mobility, Self-Care, Usual Activities, Pain/Discomfort, and Anxiety/Depression), each with three levels of functioning (no problems, some problems, and unable to/extreme problems). This health state classification describes 243 unique health states that are often reported as vectors ranging from 11111 (full health) to 33333 (worst health). Societal value sets have been derived from population-based valuation studies around the world that, when applied to the health state vectors, result in preference-based index values that typically range from states worse than dead (<0), to 1 (full health), anchoring dead at 0. In addition, the EQ-5D includes an EQ-VAS where own health “today” is rated on a scale from 0 (worst imaginable health) to 100 (best imaginable health).

In developing the 5L, the five-dimensional structure of the 3L was retained, but the descriptors within each dimension were adapted to a 5-level system based on qualitative and quantitative studies conducted by the EuroQol group [19]. The labels for 5L followed the format no problems, slight problems, moderate problems, severe problems, and unable to/extreme problems for all dimensions. For Mobility, the description of “confined to bed” was changed to “unable to walk about.” Additionally, for Usual Activities, the word “performing” was changed to “doing” (English for UK version). The official EQ-5D-3L and EQ-5D-5L language versions for each country were used.

For the purposes of the current study, respondents also rated their own health “today” on five dimension-specific rating scales, one for each of the EQ-5D dimensions. Each scale consisted of a horizontal hash-marked line (from 0 to 100) with corresponding numbers (0, 10, 20, …, 100). The descriptive anchors at each end of the scales were the same anchors as used in the 3L and 5L, that is, no problems and unable to/extreme problems.

Convergent validity was assessed by comparing the 3L and 5L dimensions to the WHO-5 Well Being questionnaire. The WHO-5 captures well-being and was developed from the World Health Organization-Ten Well-Being Index [24, 25]. It was conceptualized as a unidimensional measure that contains five positively worded items: “I have felt cheerful and in good spirits”; “I have felt calm and relaxed”; “I have felt active and vigorous”; “I woke up feeling fresh and rested”; and “My daily life has been filled with things that interest me,” all operationalized using a six-point Likert scale ranging from 0 (not present) to 5 (constantly present). A sum-score can be calculated as a summary measure.

### Analysis

Feasibility was assessed by calculating the number of missing values for 3L and 5L. The ceiling of the EQ-5D was defined as the proportion of respondents scoring no problems on any of the five dimensions, that is, the proportion of respondents scoring 11111. Under the assumption that the majority of patients should have at least some problem on at least one of the EQ-5D dimensions, we expect the ceiling to be lower for 5L compared to 3L. An absolute reduction when going from 3L to 5L was calculated, but since the ceiling was very small in some patient groups, a percentage reduction was also calculated: (ceiling_3L_ − ceiling_5L_)/ceiling_3L_.

#### Redistribution properties of the 3L to 5L extension

Redistribution properties and (in)consistency of responses were evaluated using criteria established in previous studies [20, 21]. An inconsistent response was defined as a 3L response followed by a 5L response that was at least two levels away. The redistribution properties of the consistent response pairs were described as proportions of the 3L–5L response pairs within each 3L response level (3L-1, 3L-2, and 3L-3) and corresponding dimension-specific rating scale values. For valid redistribution, dimension-specific rating scale values should be increasing when going from the “healthiest” response pair (3L-1 paired with 5L-1) to the most extreme response pair (3L-3 paired with 5L-5).

#### Discriminatory power

The Shannon index and the Shannon Evenness index were used to assess discriminatory power. Originating from the field of information theory, the Shannon index has been widely used in ecological studies as a measure of biodiversity and in molecular biology as a measure of the information content of DNA molecules [26–28]. Previous research showed Shannon’s methodology to be useful in assessing discriminatory power in health state classifications [20, 21, 23, 29, 30]. In the present study, we estimated discriminatory power for each dimension separately. The Shannon index is defined as:

$$ H^{\prime} = - \sum\limits_{i = 1}^{L} {p_{i} \log_{2} } p_{i} $$

where *H*′ represents the absolute amount of informativity captured, *L* is the number of levels, and *p*_*i*_ = *n*_*i*_ */N*, the proportion of observations in the *i*th level (*i* = 1, …, *L*), where *n*_*i*_ is the observed number of scores (responses) in level *i* and *N* is the total sample size [31]. The higher the index *H*′ is, the more information is captured by the system. In the case of a uniform (rectangular) distribution (i.e., *p*_*i*_ = p* for all *i*), the optimal amount of information is captured and *H*′ has reached its maximum (*H*′_max_) which equals log_2_ *L*. If the number of levels (*L*) is increased, *H*′_max_ increases accordingly, but *H*′ will only increase if the newly added levels are actually used. Shannon Evenness index (*J*′) exclusively reflects the evenness (rectangularity) of a distribution, regardless of the number of levels. Shannon Evenness index (*J*′) is defined as: *J*′ = *H*′*/H*′_max_. The Shannon indices are calculated by dimension and also by instrument as a whole, treating each health state vector as a unique category.

The Shannon indices are purely descriptive measures of the discriminatory power of a classification system and have no relation to the content, meaning, or clinical relevance of what the instrument aims to measure. Both the Shannon index and the Shannon Evenness index are needed to make a useful interpretation of the discriminatory power of a measurement scale. Consider any 3L and 5L dimension: Clearly, the 5L has more discriminatory potential. However, if the extra levels are not used, the *H*′ value will be the same in both dimensions. Therefore, the Shannon Evenness index *J*′, which will be lower, is needed to express the loss in potential of the 5-level dimension. Conversely, when both the 3L and 5L show rectangular distributions, the *J*′ value will be the same. In this case, *H*′ is needed to express the better discriminatory performance of the 5L. We expected *H*′ to increase and *J*′ to marginally decrease at most.

#### Convergent validity

Convergent validity between the 3L and 5L dimensions and the WHO-5 items was assessed using Spearman rank order coefficients (Spearman’s rho), including a comparison with the WHO-5 sum-score. We hypothesized correlations to be highest for WHO-5 items with Anxiety/Depression. Convergence of 3L and 5L with dimension-specific rating scales was also assessed.

#### Known-groups validity

Known-groups validity was tested for all 3L and 5L dimensions in regard to age, education, and smoking status. Tests for age-groups (18–24, 35–44, 45–54, 55–64, 65–74, and 75+) and education were performed using Spearman rank order coefficients, and smoking status (never smoked, ex-smoker, and current smoker) was assessed with the Kruskall–Wallis *H* statistic. Education was included in three substudies (England, Denmark, and Scotland) and was recoded into three levels (1 = primary/lower secondary; 2 = secondary/vocational; 3 = higher/college). In regard to known-groups validity, we expected a lower reported health status for respondents with increasing age, lower education, and respondents who smoke or have smoked. In order to take possible clustering effects into account, we applied a set of statistical techniques developed for nonparametric statistics for clustered data, with country as cluster variable [32, 33].

The study data were analyzed centrally using PASW version 18.0.0 and R version 2.15.2.

## Results

In total, 3,919 respondents completed both the 3L and 5L (Table 1). The overall cohort was 52 % female and had a mean age of 51.9 (standard deviation (SD) 20). A mean (SD) EQ-VAS score of 64 (23) was observed, ranging from 41 (30) for Parkinson’s disease to 79 (16) for the student sample. For 5L, 683 unique health states were observed (22 % of the total number of theoretically possible health states) versus 124 for 3L (51 % of the total).

**Table 1:** Characteristics and descriptive results of study sample by country and patient group

Country	Population	*N*	% female	Mean age (years)	Mean EQ-VAS (SD)
Denmark	Diabetes	239	45	52.9	74 (19)
	Orthopedic accident	94	34	37.8	79 (23)
	Rheumatoid arthritis	35	73	60.5	60 (25)
England	ADHD	69	54	34.3	63 (21)
	Arthritis	250	44	57.7	66 (20)
	Back pain	70	57	47.2	52 (19)
	COPD	125	37	60.8	57 (21)
	Depression	250	56	42.4	62 (21)
	Diabetes	45	58	50.8	69 (20)
	Myocardial infarction	75	27	56.7	63 (20)
	Parkinson’s disease	32	44	49.8	66 (22)
	Stroke	85	39	57.4	53 (24)
Italy	Liver disease	645	35	56.7	70 (21)
Netherlands	Kidney dialysis	49	41	61.7	62 (21)
	Personality disorders	384	67	31.7	59 (18)
Poland	Stroke	529	49	69.9	52 (26)
	Student population	443	79	22.1	79 (16)
Scotland	Asthma	21	57	72.8	64 (18)
	Cardiovascular disease	176	54	71.4	60 (21)
	COPD	196	62	70.1	58 (21)
	Multiple sclerosis	15	53	63.9	52 (21)
	Parkinson’s disease	5	60	63.0	41 (30)
	Rheumatoid arthritis	87	71	69.4	56 (22)
Total		3,919	52	51.9	64 (23)

*ADHD* attention-deficit/hyperactivity disorder, *COPD* chronic obstructive pulmonary disease, *EQ*-*VAS* EQ-5D visual analogue scale, where respondent rated own health on a scale from 0 (worst imaginable health) to 100 (best imaginable health)

Respondents were classified into nine different subgroups that included cardiovascular disease (*n* = 251), COPD/asthma (*n* = 342), depression (*n* = 250), diabetes (*n* = 284), liver disease (*n* = 645), personality disorders (*n* = 384), rheumatoid arthritis/arthritis (*n* = 372), stroke (*n* = 614), and students (*n* = 443). Less prevalent conditions listed in Table 1 were collapsed into an “other conditions” category (*n* = 334). The average number of unique health states by subgroup was 49 for 3L ranging from 16 (student population) to 73 (stroke patients), and 158 for 5L ranging from 49 (student population) to 280 (stroke cohort).

Missing values ranged from 43 for Mobility (1.1 %) to 57 for Pain/Discomfort (1.5 %) for 3L and from 19 for Mobility (0.5 %) to 37 for Usual Activities (0.9 %) for 5L. Missing values were on average 0.8 % for 5L and 1.3 % for 3L, indicating good feasibility for both instruments.

Cross tabulations of responses to the 3L and 5L, which include all data, showed that participants reported a wide range of level of health within each of the EQ-5D dimensions (Table 2). The areas shaded gray in Table 2 show the inconsistent responses. The number of inconsistencies was highest in Pain/Discomfort (*n* = 130; 3.4 %) and lowest in Mobility (*n* = 82; 2.1 %). The average proportion of inconsistencies by dimension was 2.9 %.

**Table 2:** Cross tabulation for EQ-5D-3L and EQ-5D-5L dimension scores (inconsistent responses are marked with italicized values)

3L	5L				
Mobility	No problems	Slight problems	Moderate problems	Severe problems	Unable to
No problems	1,941	121	*16*	*1*	*4*
Some problems	*32*	588	598	393	*23*
Confined to bed	*1*	*1*	*4*	30	112

3L	5L				
Self-Care	No problems	Slight problems	Moderate problems	Severe problems	Unable to
No problems	2,653	83	*13*	*5*	*0*
Some problems	*48*	425	321	110	*6*
Unable to	*3*	*5*	*6*	35	141

3L	5L				
Usual Activities	No problems	Slight problems	Moderate problems	Severe problems	Unable to
No problems	1,527	167	*22*	*9*	*0*
Some problems	*49*	686	676	277	*16*
Unable to	*5*	*7*	*24*	140	242

3L	5L				
Pain/Discomfort	None	Slight	Moderate	Severe	Extreme
None	1,251	211	*21*	*6*	*2*
Moderate	*67*	895	869	244	*9*
Extreme	*1*	*5*	*19*	160	83

3L	5L				
Anxiety/Depression	None	Slight	Moderate	Severe	Extreme
None	1,466	220	*31*	*10*	*3*
Moderate	*46*	890	731	165	*7*
Extreme	*1*	*4*	*17*	163	94

Table 3 shows 3L and 5L dimension responses for the eight patient groups and the student cohort. Overall, 5L responses show a good spread for most dimensions and patient samples, revealing the benefit of the extra levels in the 5L. The responses in Mobility show the effect of changing the most extreme level from “confined to bed” to “unable to walk about,” as respondents make better use of the 5L scale.

**Table 3:** Dimension responses for EQ-3D-3L and EQ-3D-5L across eight patient groups and a student cohort

	Level	Mobility				Self-Care				Usual Activities				Pain/Discomfort				Anxiety/Depression			
		3L		5L		3L		5L		3L		5L		3L		5L		3L		5L	
		*N*	%	*N*	%	*N*	%	*N*	%	*N*	%	*N*	%	*N*	%	*N*	%	*N*	%	*N*	%
Cardiovascular disease	1	70	*28*	56	*22*	145	*58*	136	*54*	75	*30*	64	*25*	74	*29*	64	*25*	126	*50*	110	*44*
	2	179	*71*	60	*24*	94	*37*	61	*24*	135	*54*	57	*23*	151	*60*	71	*28*	111	*44*	70	*28*
	3	2	*1*	74	*29*	12	*5*	35	*14*	41	*16*	67	*27*	26	*10*	61	*24*	14	*6*	51	*20*
	4	–	–	56	*22*	–	–	12	*5*	–	–	42	*17*	–	–	45	*18*	–	–	14	*6*
	5	–	–	5	*2*	–	–	7	*3*	–	–	21	*8*	–	–	10	*4*	–	–	6	*2*
COPD/Asthma	1	85	*25*	72	*21*	203	*59*	192	*56*	89	*26*	76	*22*	79	*23*	76	*22*	177	*52*	163	*48*
	2	255	*75*	80	*23*	129	*38*	70	*20*	213	*62*	91	*27*	214	*63*	88	*26*	143	*42*	81	*24*
	3	2	*1*	94	*27*	10	*3*	52	*15*	40	*12*	87	*25*	49	*14*	105	*31*	22	*6*	74	*22*
	4	–	–	90	*26*	–	–	19	*6*	–	–	66	*19*	–	–	60	*18*	–	–	20	*6*
	5	–	–	6	*2*	–	–	9	*3*	–	–	22	*6*	–	–	13	*4*	–	–	4	*1*
Depression	1	165	*66*	154	*62*	205	*82*	204	*82*	119	*48*	113	*45*	107	*43*	82	*33*	46	*18*	33	*13*
	2	84	*34*	54	*22*	44	*18*	21	*8*	118	*47*	72	*29*	121	*48*	88	*35*	175	*70*	89	*36*
	3	1	*0*	24	*10*	1	*0*	21	*8*	13	*5*	37	*15*	22	*9*	48	*19*	29	*12*	80	*32*
	4	–	–	17	*7*	–	–	4	*2*	–	–	25	*10*	–	–	24	*10*	–	–	32	*13*
	5	–	–	1	*0*	–	–	0	*0*	–	–	3	*1*	–	–	8	*3*	–	–	16	*6*
Diabetes	1	189	*68*	179	*64*	232	*83*	231	*83*	172	*61*	162	*58*	129	*47*	115	*42*	189	*68*	173	*62*
	2	89	*32*	53	*19*	47	*17*	36	*13*	95	*34*	69	*25*	135	*49*	93	*34*	87	*31*	71	*26*
	3	0	*0*	26	*9*	0	*0*	8	*3*	13	*5*	28	*10*	13	*5*	41	*15*	3	*1*	25	*9*
	4	–	–	21	*8*	–	–	4	*1*	–	–	13	*5*	–	–	23	*8*	–	–	7	*3*
	5	–	–	0	*0*	–	–	0	*0*	–	–	6	*2*	–	–	5	*2*	–	–	1	*0*
Liver disease	1	457	*74*	465	*73*	542	*88*	568	*89*	425	*68*	428	*68*	367	*60*	365	*58*	346	*56*	347	*55*
	2	163	*26*	103	*16*	73	*12*	42	*7*	183	*29*	106	*17*	233	*38*	151	*24*	249	*40*	166	*26*
	3	1	*0*	53	*8*	3	*0*	21	*3*	14	*2*	69	*11*	15	*2*	94	*15*	22	*4*	97	*15*
	4	–	–	17	*3*	–	–	4	*1*	–	–	22	*3*	–	–	19	*3*	–	–	19	*3*
	5	–	–	1	*0*	–	–	2	*0*	–	–	6	*1*	–	–	3	*0*	–	–	5	*1*
Personality disorder	1	324	*85*	320	*84*	357	*94*	357	*93*	120	*31*	98	*26*	168	*44*	137	*36*	64	*17*	51	*13*
	2	58	*15*	39	*10*	24	*6*	21	*5*	228	*60*	85	*22*	197	*52*	132	*34*	217	*57*	82	*21*
	3	1	*0*	21	*5*	0	*0*	3	*1*	33	*9*	119	*31*	17	*4*	85	*22*	100	*26*	119	*31*
	4	–	–	2	*1*	–	–	1	*0*	–	–	70	*18*	–	–	26	*7*	–	–	105	*27*
	5	–	–	1	*0*	–	–	0	*0*	–	–	10	*3*	–	–	3	*1*	–	–	25	*7*
RA/Arthritis	1	106	*29*	83	*22*	235	*64*	223	*60*	106	*29*	81	*22*	45	*12*	26	*7*	222	*60*	190	*51*
	2	263	*71*	115	*31*	132	*36*	84	*23*	232	*63*	131	*36*	282	*76*	123	*33*	134	*36*	100	*27*
	3	0	*0*	101	*27*	3	*1*	43	*12*	32	*9*	94	*25*	43	*12*	135	*37*	14	*4*	54	*15*
	4	–	–	67	*18*	–	–	17	*5*	–	–	46	*12*	–	–	73	*20*	–	–	18	*5*
	5	–	–	3	*1*	–	–	2	*1*	–	–	17	*5*	–	–	12	*3*	–	–	7	*2*
Stroke	1	133	*22*	121	*20*	201	*33*	190	*31*	118	*20*	108	*18*	122	*20*	117	*19*	141	*23*	122	*20*
	2	359	*59*	117	*19*	263	*44*	122	*20*	309	*51*	127	*21*	428	*71*	148	*25*	416	*69*	213	*35*
	3	115	*19*	160	*26*	139	*23*	117	*19*	176	*29*	141	*23*	50	*8*	212	*35*	46	*8*	169	*28*
	4	–	–	113	*19*	–	–	60	*10*	–	–	95	*16*	–	–	100	*17*	–	–	79	*13*
	5	–	–	99	*16*	–	–	118	*19*	–	–	133	*22*	–	–	26	*4*	–	–	22	*4*
Students	1	434	*98*	428	*97*	442	*100*	442	442	398	*90*	376	*85*	297	*67*	268	*60*	246	*56*	190	*43*
	2	9	*2*	12	*3*	1	*0*	0	1	44	*10*	48	*11*	145	*33*	143	*32*	192	*43*	173	*39*
	3	0	*0*	2	*0*	0	*0*	1	*0*	1	*0*	15	*3*	1	*0*	29	*7*	5	*1*	55	*12*
	4	–	–	1	*0*	–	–	0	*0*	–	–	3	*1*	–	–	3	*1*	–	–	21	*5*
	5	–	–	0	*0*	–	–	0	*0*	–	–	1	*0*	–	–	0	*0*	–	–	4	*1*

*COPD* chronic obstructive pulmonary disease, *RA* rheumatoid arthritis

Redistribution from 3L to 5L using the dimension-specific rating scales as reference showed valid results for all 35 3L–5L (consistent) level combinations, as the mean rating scale scores decreased when going from the healthiest subgroup to the most disabled subgroup, regardless of dimension (Table 4). Proportions (% by level) show considerable variation across dimensions. For the 3L–1 subgroups of each dimension, there was always a higher proportion in 5L–1 than in 5L–2. The most skewed relative frequency distribution was in Self-Care (97/3) and the least in Pain/Discomfort (86/14). The 3L–2 subgroups showed variable proportions per dimension; the most evenly spread proportion was in Mobility (37/38/25) and the most unevenly spread in Anxiety/Depression (50/41/9). The 5L–4 scores always corresponded with the lowest proportion for 3L–2. The 3L–3 scores corresponded with the largest proportion in 5L–5 for the first three dimensions, but were associated with more 5L–4 scores in the case of Pain/Discomfort and Anxiety/Depression.

**Table 4:** Redistribution properties from EQ-5D-3L to EQ-5D-5L: consistent responses

Dimension	3L	*n*	% by dimension	5L	*n*	% by level	Rating scale mean^a^
Mobility	1	2,083	53.9	1	1,941	94.1	96.8
				2	121	5.9	84.5
	2	1,634	42.3	2	588	37.2	70.0
				3	598	37.9	52.4
				4	393	24.9	32.1
	3	148	3.8	4	30	21.1	16.6
				5	112	78.9	3.1
Self-Care	1	2,754	71.5	1	2,653	97.0	98.0
				2	83	3.0	81.6
	2	910	23.6	2	425	49.6	68.6
				3	321	37.5	49.4
				4	110	12.9	32.9
	3	190	4.9	4	35	19.9	18.2
				5	141	80.1	6.1
Usual Activities	1	1,725	44.8	1	1,527	90.1	96.7
				2	167	9.9	86.8
	2	1,704	44.3	2	686	41.9	72.4
				3	676	41.2	53.1
				4	277	16.9	36.9
	3	418	10.9	4	140	36.6	20.1
				5	242	63.4	8.8
Pain/Discomfort	1	1,491	38.8	1	1,251	85.6	95.7
				2	211	14.4	84.4
	2	2,084	54.2	2	895	44.6	72.5
				3	869	43.3	54.5
				4	244	12.2	37.2
	3	268	7.0	4	160	65.8	21.8
				5	83	34.2	13.0
Anxiety/Depression	1	1,730	45.0	1	1,466	87.0	97.2
				2	220	13.0	84.6
	2	1,839	47.8	2	890	49.8	66.4
				3	731	40.9	50.0
				4	165	9.2	38.3
	3	279	7.3	4	163	63.4	28.5
				5	94	36.6	13.1

^a^Dimension-specific rating scale values were only available for a subset of respondents (without the student and liver disease samples); respondents rated own level of health by dimension on scales from 0 (worst) to 100 (best)

The ceiling by disease subgroup and by country is shown in Table 5. The reduction in ceiling going from 3L to 5L varied considerably over subgroups and countries, ranging from an absolute reduction of 1.1 % for stroke patients to 12.6 % for the student cohort. Percentage reduction ranged from 7.1 % for the Danish population to 49.0 % for the Dutch population. On average, the ceiling was reduced from 20.2 % (3L) to 16.0 % (5L), an absolute reduction of 4.2 % and a percentage reduction of 20.8 %. Overall, the ceiling was reduced the least for the Danish and Italian population samples.

**Table 5:** Ceiling for EQ-5D-3L and EQ-5D-5L in nine subgroups and six countries

	Ceiling 3L (% 11111)	Ceiling 5L (% 11111)	Absolute reduction (%)	Percentage reduction (%)
*Subgroup*				
Cardiovascular disease	13.1	8.0	5.2	39.4
COPD/Asthma	8.5	7.0	1.5	17.2
Depression	12.0	6.4	5.6	46.7
Diabetes	33.9	28.3	5.7	16.7
Liver disease	38.5	35.7	2.8	7.2
Personality disorder	7.7	3.9	3.8	48.8
RA/Arthritis	6.5	1.9	4.6	70.8
Stroke	7.1	6.0	1.1	15.0
Students	47.0	34.3	12.6	26.9
*Country*				
Denmark	32.8	30.4	2.3	7.1
England	10.0	5.7	4.3	43.0
Italy^a^	38.5	35.7	2.8	7.2
Netherlands	7.8	4.0	3.8	49.0
Poland	23.6	17.6	6.0	25.4
Scotland	9.6	6.0	3.6	37.5
Total	20.2	16.0	4.2	20.8

*COPD* chronic obstructive pulmonary disease, *RA* rheumatoid arthritis

^a^Identical to liver disease cohort

Absolute discriminatory power (Shannon index) showed a substantial gain in information richness by using the 5L classification system for all dimensions and the overall classification system (*H*′_5L_/*H*′_3L_): Mobility (1.89/1.19); Self-Care (1.42/1.05); Usual Activities (2.08/1.39); Pain/Discomfort (2.01/1.28); Anxiety/Depression (1.96/1.30); and overall (4.8/7.2). Relative discriminatory power (Shannon Evenness index) improved slightly for most dimensions and the overall system (*J*′_5L_/*J*′_3L_): Mobility (0.81/0.75); Self-Care (0.61/0.66); Usual Activities (0.89/0.88); Pain/Discomfort (0.87/0.81); Anxiety/Depression (0.85/0.82); and overall (0.62/0.61). On average, absolute discriminatory power improved considerably with 5L (mean 1.87 for 5L versus 1.24 for 3L), and relative discriminatory power improved slightly (mean 0.81 for 5L versus 0.78 for 3L), confirming our hypothesis.

There is evidence of convergent validity of 3L and 5L with the WHO-5 (Table 6). All Spearman rank order coefficients for 3L and 5L comparisons with the five WHO-5 items were significant (*p* < 0.001). Correlations were highest for Anxiety/Depression, especially with feeling in good spirits (3L = 0.55; 5L = 0.57) and feeling calm and relaxed (3L = 0.61; 5L = 0.61), as expected. High correlations were also found between Mobility, Self-Care, and Usual Activities with feeling active and vigorous (“Energy”), showing correlation coefficients over 0.50 except for 3L Mobility (0.43). The 5L dimensions demonstrated slightly better convergent validity compared with 3L, with the largest difference observed for Mobility. Correlations with the WHO-5 sum-scores were 0.49 for 3L on average (ranging from 0.39 for Mobility to 0.58 for Anxiety/Depression) and 0.53 for 5L on average (ranging from 0.48 for Pain/Discomfort to 0.58 for Anxiety/Depression). Convergence of 3L and 5L with dimension-specific rating scales improved slightly with 5L over 3L (mean Spearman’s rho 0.80 versus 0.77, respectively).

**Table 6:** Convergent validity: 3L and 5L dimensions with WHO-5 (Spearman rank order coefficients*)

WHO-5	Good spirits		Relaxed		Energy		Fresh and rested		Interested in things	
EQ-5D	3L	5L	3L	5L	3L	5L	3L	5L	3L	5L
Mobility	0.27	0.39	0.24	0.34	0.43	0.54	0.30	0.33	0.27	0.39
Self-Care	0.39	0.44	0.37	0.39	0.51	0.53	0.37	0.39	0.40	0.39
Usual Activities	0.40	0.40	0.36	0.34	0.54	0.59	0.36	0.40	0.39	0.41
Pain/Discomfort	0.35	0.37	0.32	0.35	0.41	0.47	0.36	0.41	0.29	0.29
Anxiety/Depression	0.55	0.57	0.61	0.61	0.39	0.40	0.43	0.43	0.42	0.42
Average	0.39	0.43	0.38	0.41	0.46	0.51	0.36	0.39	0.35	0.38

Substudy for England only (*N* = 1001)

* All *p* < 0.001

Results for known-groups validity are shown in Table 7 and confirmed our hypotheses. All 3L and 5L correlations with age are significant and in the expected direction, showing increased reported problems for each dimension with increasing age, except for Anxiety/Depression which shows slightly less reported problems with increasing age. Results for education were similar, showing significantly less reported problems with higher education, except for Anxiety/Depression (nonsignificant). Correlations were generally similar for 5L and 3L. Kruskall–Wallis tests showed significant results for all dimensions except 3L Pain/Discomfort. The percentage proportions showed increasing reported problems going from nonsmokers to ex-smokers and smokers as expected. The analyses for clustering showed that for age all comparisons were still significant, although the *p* values were higher (range 0.004–0.041). For education and smoking cluster analyses resulted in nonsignificant results for all 3L and 5L dimensions. When performing analyses for the separate countries on education, Scotland showed significant results for all 3L and 5L dimensions, England showed significant for all 3L and 5L dimensions except Self-Care and Anxiety/Depression, and Denmark showed nonsignificant results for all 3L and 5L dimensions. In regard to smoking, for all 3L and 5L dimensions England showed significant results and Scotland showed nonsignificant results.

**Table 7:** Known-groups validity: 3L and 5L with socio-demographic variables^a^

Demographic variable	Age-groups (Spearman’s rho)		Education (Spearman’s rho)		Smoking^b^ (*p* value)	
	3L	5L	3L	5L	3L	5L
Mobility	0.44**	0.45**	−0.16**	−0.20**	<.001	<.001
Self-Care	0.32**	0.33**	−0.13**	−0.13**	<.001	<.001
Usual Activities	0.28**	0.27**	−0.15**	−0.17**	<.001	<.001
Pain/Discomfort	0.23**	0.24**	−0.15**	−0.14**	0.068	<.001
Anxiety/Depression	−0.04*	−0.06**	−0.04	−0.04	<.001	<.001

* *p* < 0.05; ** *p* < 0.001

^a ^Education was included only in Denmark, England, and Scotland (*n* = 1,869); smoking status was included in England and Scotland (*n* = 1,501)

^b^Kruskall–Wallis *H*

## Discussion

The aim of this study was to assess the performance of the 5L, in comparison with the 3L, in terms of feasibility (missing values), redistribution properties, ceiling, discriminatory power (Shannon indices), and convergent validity. The 5L performed similar in terms of feasibility, showed increased discriminatory power, slightly improved convergent validity, and similar known-groups validity. Redistribution was confirmed, and the ceiling was reduced with 5L.

The frequency proportions of the redistribution showed varying distributions over the dimensions. As expected, the healthiest subgroup within dimensions (3L-1 paired with 5L-1) always showed the largest proportion, since many (treated) health conditions display no symptoms or problems on a particular dimension no matter how refined the response scale. In all dimensions, the 3L-3 and 5L-4 response pair proportion was large (≥ 20 %). This supports the inclusion of a fourth level at this position, as many respondents opted for “severe problems” on 5L compared to “extreme problems” on 3L–3. The same applies to the response pair 3L-2 and 5L-2, where many respondents opted for “slight problems” on 5L compared to “some/moderate problems” on 3L–3, thus supporting the inclusion of a second level at this position. The response pair 3L-2 and 5L-4 was smaller than expected for some dimensions, that is, 9 % for Anxiety/Depression, 12 % for Pain/Discomfort, and 13 % for Self-Care. It would seem that for these dimensions, “some” or “moderate” problems on 3L are better covered by “slight” or “moderate” problems on 5L, rather than by “severe” problems on 5L.

Due to the lower threshold (i.e., presence of level 5L–2, “slight” problems), we expected a lower ceiling in the 5L version. There was indeed a significant reduction in the ceiling for most patient groups. When the absolute reduction is low but the ceiling is also low, it can be more useful to look at the percentage reduction. This revealed a considerable reduction in the current study (e.g., for COPD/asthma, personality disorder, RA/arthritis, and stroke). For some countries both the absolute and percentage reduction were rather low, however, such as in the Danish and Italian patient samples. It is possible that these are “true” findings: When respondents have no problems on the five dimensions, they will report “no problems” no matter how many levels were added. For the Danish sample, this was supported by the relatively good health status of the participating patients, especially those with diabetes who comprised the main part of the sample. Thus, 39 % of the Danish patients with diabetes reported that the severity of their condition was “mild,” 41 % had no diabetic complications, and 32 % rated their self-perceived health as either “excellent” or “very good.” The Italian sample consisted wholly of liver disease patients with few problems on any dimension regardless of whether the 3L or 5L version was used. Responses for some of the subgroups in this sample, including chronic hepatitis, cirrhosis, and patients who received liver transplantation, might be influenced by effective coping mechanisms to deal with these long-term conditions.

Extending the EQ–5D descriptive system to a five-level version resulted in higher absolute discriminatory power than for the three-level version in all dimensions, as expected. Surprisingly, relative discriminatory power (evenness) did not deteriorate in the 5L but was slightly better than for the 3L version. The high evenness score in all 5L dimensions indicated that the extra levels were used efficiently. Convergent validity with WHO-5 improved with 5L, especially for Mobility, which might be caused by changing the 3L level “confined to bed” to “unable to walk about.” Known-groups validity was confirmed for both 5L and 3L, showing similar results. Cluster analyses had no impact on the analyses for age but brought the results for education and smoking into question. Separate analyses for each country confirmed the hypothesis for all countries again, except for Denmark where education had no impact and for Scotland where smoking had no impact. For Denmark this is likely due to a power issue since the mean level scores all point in the right direction, and the relatively healthy Danish sample shows a rather homogeneous distribution, making it harder to find statistically significant differences. For Scotland possibly the old age of the respondents and the low reported health status might mask the effects of smoking.

The results of this study provide evidence of the validity of the EQ-5D-5L in a range of patient groups across six countries. Not all measurement properties were tested in the current study. The Korean version of the EQ-5D-5L has proved to be reliable in cancer patients [23], but reliability still needs to be determined for other language versions and other patient groups. Furthermore, responsiveness to health changes over time still needs to be assessed. A limitation of the current study is that since 5L was always tested first, there may have been an order effect. The order effect could account for the slightly higher proportion of missing values for the 3L. A further limitation is that since the study was mainly conducted in patient population settings, it was not possible to calculate and apply sampling weights.

Alongside the descriptive classification system, an important aspect of the EQ-5D is the availability of index-based value sets. Valuation studies for the 5L are in progress around the world and are likely to be published in the near future. Until these studies are finalized, index values for 5L based on the 3L value sets are available using a mapping approach, described in detail by van Hout et al. (2012) and on the EuroQol Web site at www.euroqol.org [34].

In conclusion, the EQ-5D-5L is a descriptive system based upon the dimensions of the EQ-5D-3L that demonstrates valid redistribution, reduced ceiling, and improved discriminatory power and convergent validity. Future studies that further examine the properties of the EQ-5D-5L in specific conditions and patient populations, particularly studies comparing the EQ-5D-5L to the EQ-5D-3L, are encouraged.
